# Correlates of Non-suicidal Self-Injury in Adolescent Psychiatric Patients in China

**DOI:** 10.3389/fpsyt.2022.864150

**Published:** 2022-06-27

**Authors:** Ji-Jie Zhang, Yin-Du Liu, Hua Zhang, Zhuo-Hui Huang, Fei Wang, Jing-Jing Yang, Shi-Bin Wang, Fu-Jun Jia, Cai-Lan Hou

**Affiliations:** ^1^The Second School of Clinical Medicine, Southern Medical University, Guangzhou, China; ^2^Guangdong Mental Health Center, Guangdong Provincial People's Hospital, Guangdong Academy of Medical Sciences, Guangzhou, China; ^3^Shantou University Medical College, Shantou, China; ^4^Dongying People's Hospital, Dongying, China

**Keywords:** non-suicidal self-injury, adolescent psychiatric patients, childhood trauma, peer bullying, romantic relationship, depression

## Abstract

**Background:**

Non-suicidal self-injury (NSSI) has attracted more and more attention from schools, mental health workers and even the whole society in China. The aim of this study was to explore influencing factors and clinical characteristics of NSSI in adolescent psychiatric patients in China, and provide valuable information for the intervention and treatment of NSSI.

**Methods:**

The sample included 157 adolescents, 114 were female (72.6%), aged 12–18 years (*M* = 15.39, SD = 1.81). Assessments were performed using Childhood Trauma Questionnaire-Short Form, the Revised Olweus Bully/Victim Questionnaire, the modified version of Adolescents Self-Harm Scale, Montgomery–Asberg Depression Rating Scale, Brief Psychiatric Rating Scale, the Young Mania Rating Scale and the Mini International Neuropsychiatric Interview. Clinical characteristics were collected from electronic medical record system.

**Results:**

Adolescent patients with NSSI experienced more adverse life events including peer bullying, childhood trauma and romantic relationship trouble. They had more times of hospitalization, higher dosage of psychotic medication, and more severe depressive symptoms.

**Conclusions:**

It is very necessary to evaluate negative life events, including childhood trauma, peer bullying etc., in adolescent patients with NSSI. It has important clinical implications for evaluating the risk factors of NSSI and giving effective intervention treatment. Timely and effective treatment of these patients' depressive symptoms will also contribute to the relief of NSSI.

## Introduction

Non-suicidal self-injury (NSSI) refers to deliberate and direct body destruction, which is not accompanied by actions that lead to the individual's death (1). NSSI has characteristics of repeated occurrence and release of negative emotion (2) which has emerged as a challenging public health problem. However, over the past 30 years, the field of NSSI research has focused primarily on Caucasian Western samples (3). Studies of NSSI-related from non-Western cultures have begun to emerge in last 10 years (4). Previous studies showed the prevalence of non-suicidal self-injury varies across studies due to some factors such as the differences in the definition and assessment tools of NSSI, culture and population (1, 5).

A study conducted in 11 European countries among adolescents showed overall lifetime prevalence of self-injury was 27.6% (5). A meta-analysis including 626 million individuals showed the lifetime prevalence of non-suicidal self-injury among preadolescents were 6.2% in community samples (6). In China, a nationwide survey (*n* = 1,908) in rural area showed 12.2% of adolescents met the suggested DSM-5 criteria for NSSI (7); and a meta-analysis (*n* = 160,348) showed the prevalence of non-suicidal self-injury was 14.5% in middle school and high school students (8).

The prevalence of NSSI has significantly increased in recent years. McManus et al. reported that the prevalence of self-reported lifetime NSSI increased from 2.4% in 2000 to 6.4% in 2014 (9). Comparing with 14.5% (2017) reported in a meta-analysis (8), a school-based study published in 2021 conducted in China showed the 1-year prevalence of NSSI was 25.2% in (10). The prevalence of NSSI among adolescents is high which attracting extensive attention in the field of education, mental health and society in China. Previous study shows adolescents experience significant pressure due to school examinations and high school entrance examination or college entrance examination in China (11), sleep deprivation and oversleeping have become increasingly prevalent in Chinese adolescent (12), which related to NSSI in some content.

Previous studies showcased a higher prevalence of NSSI in clinical (50%) than in community samples (17–18%) among adolescent psychiatric patients (13). However, most of the studies concerning the characteristics, risk factors, and mechanism of NSSI among adolescent come from the general population (14). Meanwhile, there is still a paucity of evidence for the exact factors and effective treatment interventions against NSSI (15). And in the current psychiatric clinic, more and more teenagers with mental disorders show self-injury behavior, which also makes the treatment and response of mental problems very difficult.

Herein, we hope to explore the influencing factors and clinical characteristics of NSSI adolescents with mental distress in China to provide valuable information for the intervention and treatment of NSSI and psychological problems.

## Methods

### Participants

Participants were recruited from the psychiatric outpatient and inpatient unit in Guangdong mental health center of Guangdong Provincial People's Hospital, situated in the Province of Guangdong, China from October to December 2020. Sample size was calculated by MedSci Sample Size tools. According to unmatched case-control study, we calculated the final sample size was 141 by prevalence of cyber bully (16) and the odds ratio of exposure (17).

### Procedure

#### Key Points of Informed Consent

The convenient sampling method was used in this survey. After obtained the written informed consent from adolescent participant and their parent/legal guardian, we will interview and evaluate the patient face-to-face. Patients were informed that they can ask the researcher to stop the interview if they feel uncomfortable during the process, or they can end the interview without any reason.

All participants were divided into the NSSI group or non-NSSI group by a psychiatrist according to the Diagnostic and statistical manual of mental disorders (DSM-5) recommended diagnostic criteria for non-suicidal self-injury.

#### Inclusion Criteria

The inclusion criteria included aged 12–18 years old, can read and understand the contents of the questionnaire, and the patients and their families are willing to participate in the interview and evaluation.

#### Exclusion Criteria

Exclusion criteria included that participant who are difficult to communicate because of their unstable mental conditions.

## Measures

### Socio-Demographic Characteristics

Using the self-report form, we collected data of years of age, gender, residence, nationality, parent's marriage and years of education. Meanwhile, we evaluated their romantic relationship by asking this question “Whether you have been troubled in a romantic relationship in the last month? Yes/No”.

### Clinical Characteristics

A self-made case report form was used to collect the clinical data of the participants from electronic medical record including diagnosis of the tenth edition of the International Classification of Diseases (ICD-10), outpatient/inpatient, whether it was the first onset, age at the first onset, number of hospitalizations, on use of mood stabilizers, dosage of antipsychotics, antidepressants, and benzodiazepines. Doses of psychotic drugs were converted into the prescribed daily dose/the defined daily dose ratio (PDD/DDD ratio) (18, 19).

All participants received an interview of the Mini International Neuropsychiatric Interview (M.I.N.I.). This tool was designed by professor Sheehan and professor Leclubier and it is a short, structured diagnostic interview used as a tool to diagnose 16 axis I (Diagnostic and Statistical Manual) DSM-IV disorders and one personality disorder (20). Shu et al. translated it to Chinese version and conducted a study on the reliability and validity in psychiatric clinic. The result shows the inter-rater and test-retest reliabilities in general were 0.94 and 0.97–1.0 (21).

### The Revised Adolescent Self-Injury Scale

The revised adolescent self-injury scale (22) was used to evaluate the frequency and severity of NSSI for last 1 year in NSSI group. The scale was compiled by Zheng et al. (23) based on The Functional Assessment of Self-Mutilation (FASM) (24) and revised by Feng et al. It includes 19 items in about 18 ways of NSSI (such as cutting, scratching, hitting or banging, carving and scraping). Each item was followed by two dimensions about frequency and severity of NSSI. The frequency was coded into a 4-level scale from 0 to 3 (0—never, 1—once for the last year, 2—twice to four times for the last year, 3—at least five times for the last year). The severity was coded into a 5-level scale from 0 to 4 (0—none, 1—mild, 2—moderate, 3—severe, 4—extremely severe). The score of single item equals the point of frequency times severity. Total score was summed by the score of single item and represented the overall seriousness. The Cronbach's alpha coefficient of this questionnaire was 0.85.

### Childhood Trauma Questionnaire-Short Form

Children's trauma experience before 12 years old was evaluated by Childhood Trauma Questionnaire-Short Form (CTQ-SF). Professor Bernstein and colleagues compiled the original English version (25) and Fu et al. translated it into Chinese (26). The CTQ-SF represents a 28-item retrospective self-report questionnaire that is divided into five subscales: emotional (EN) and physical neglect (PN), emotional (EA), sexual (SA) and physical abuse (PA) (27). The frequency was coded into 5-level scale from 1 to 5 (1–never true, 2-rarely true, 3–sometimes true, 4–often true, 5–very often true). The Cronbach's alpha coefficient of this questionnaire was 0.82 (28).

### The Revised Olweus Bully/Victim Questionnaire

Peer bullying was evaluated by the Revised Olweus Bully/Victim Questionnaire (OBVQ-R) for last 1 year. The original English version of the OBVQ was developed in 1983 by Olweus and revised in 1996, and Zhang et al. translated it into Chinese in 1999. The complete OBVQ-R is a 42-item self-report questionnaire including five subscales. According to the purpose of the study, the part of being bullied were used. The questionnaire incorporated seven types of bullying including calling of people with mean names or teasing; exclusion; hitting, kicking and pushing; actions like taking money or damaging belongings; threatening; and making racial comments while the last one was cyberbullying. It is a 5-level rating scale (0—bullying has not happened. 1—only once or twice within last year. 2-−2–3 times for a month. 3—once a week. 4—several times per week). The Cronbach's alpha coefficient of this questionnaire was 0.82 (29).

### Psychological Symptoms Assessment

Montgomery–Asberg Depression Rating Scale (MADRS) (30) was used to measure the severity of the depressive episodes. Higher scores reflecting severer symptoms. A study on Chinese version resulted that the Cronbach's alpha coefficient of this questionnaire was 0.84 (31). Psychotic symptoms in patients were measured with the 18-item Brief Psychiatric Rating Scale (BPRS) (32). The BPRS Chinese edition has good reliability and validity (33). The Young Mania Rating Scale (YMRS) was used to evaluate manic symptoms. The scale's Cronbach's Alpha coefficient was 0.93 (34).

### Statistical Analysis

In this study, Pearson chi-square tests and independent-samples *t*-test as well as one-way analysis of variance (ANOVA) as appropriate to evaluate the differences in socio-demographic and clinical information between the NSSI group and non-NSSI group. The differences of scores on scales were evaluated by *t*-test. Factors showed significant level in univariate analysis were entered into the logistic regression. All analyses were conducted with the use of SPSS software version 20.0. Two-sided *P*-value of < 0.05 was considered statistically significant in the study.

## Results

A total of 164 adolescent patients were recruited, of which 7 did not complete the evaluation of the scale. Finally, 157 completed the interview and evaluation, which were included in the final analysis. The response rate was 95.73%.

Table 1 shows the socio-demographic, clinical and psychological characteristics of NSSI group and Non-NSSI group (patients without NSSI).

**Table 1:** Socio-demographic, clinical and psychological characteristics of NSSI group and Non-NSSI group (patients without NSSI).

	**Overall (*****n*** **=** **157)**		**NSSI (*****n*** **=** **80)**		**Non-NSSI (*****n*** **=** **77)**		**Statistics**		
	***N***	**%**	***N***	**%**	***N***	**%**	**χ^2^^a^**	**df**	***P***
Male	43	27.4	17	21.3	26	33.8	3.09	1	0.07
Ethnic han	152	96.8	78	97.5	74	96.1	0.24	1	0.61
Urban	134	85.4	70	87.5	64	83.1	0.60	1	0.43
Trouble in romantic relationship	30	19.1	21	26.3	9	11.7	5.38	1	**0.02**
Divorced (parents)	32	20.4	18	22.5	14	18.2	0.45	1	0.50
Outpatient	103	65.6	42	52.5	61	79.2	12.41	1	**<0.001**
First-episode patients	95	60.5	42	52.5	53	68.8	4.37	1	**0.03**
On mood-stabilizer	50	31.8	34	42.5	16	20.8	8.53	1	**<0.001**
	***M***	**SD**	**mean**	**SD**	**mean**	**SD**	**t/F** ^**b**^	**df**	***P***
Age (years)	15.39	1.81	15.45	1.75	15.34	1.88	0.38	155	0.69
Education (years)	9.61	1.76	9.63	1.81	9.60	1.73	0.09	155	0.92
First-episode age (years)	13.82	2.54	13.68	2.49	13.97	2.60	−0.72	155	0.47
Hospitalization times	0.59	0.79	0.80	0.89	0.36	0.60	3.60	139	**<0.001**
CTQ-SF total	48.20	12.69	52.00	12.72	44.26	11.47	3.99	155	**<0.001**
CTQ-SF EA	10.55	4.45	11.89	4.84	9.16	3.51	4.05	144	**<0.001**
CTQ-SF PA	7.25	3.39	7.86	3.84	6.62	2.72	2.33	142	**0.02**
CTQ-SF SA	6.24	2.84	6.38	2.74	6.10	2.95	0.59	153	0.55
CTQ-SF EN	14.98	4.57	16.04	4.20	13.88	4.70	3.02	155	**0.003**
CTQ-SF PN	9.18	2.99	9.84	3.14	8.49	2.68	2.87	155	**0.005**
OBVQ-R total	5.17	5.87	6.94	6.79	3.32	4.00	4.07	128	**<0.001**
MADRS total	17.74	10.15	20.55	9.62	14.82	9.91	3.67	155	**<0.001**
YMRS total	2.63	4.24	3.14	5.26	2.10	2.77	1.53	155	0.12
BPRS total	28.08	7.65	29.34	7.76	26.77	7.38	2.13	155	0.035
Antipsychotic PDD/DDD	0.33	0.60	0.49	0.73	0.16	0.36	3.58	155	**<0.001**
SAG PDD/DDD	0.33	0.60	0.49	0.73	0.16	0.36	3.64	116	**<0.001**
Antidepressant PDD/DDD	0.91	1.09	1.02	1.14	0.79	1.03	1.28	155	0.20
Benzodiazepine PDD/DDD	0.12	0.24	0.19	0.28	0.06	0.17	3.46	155	**<0.001**

*Bold values: P < 0.05; BPRS, brief psychiatric rating scale; OBVQ-R, the revised olweus bully/victim questionnaire; CTQ-SF, childhood trauma questionnaire-short form; DDD, defined daily dose; EA, emotional abuse; EN, emotional neglect; MADRS, montgomery–asberg depression rating scale; PA, physical abuse; PDD, prescribed daily dose; PN, physical neglect; SA, sexual abuse; SAG, second generation antipsychotics; YMRS, young mania rating scale;*

a *Pearson chi-square tests;*

b *independent-samples t-test or one-way analysis of variance*.

A total of 157 adolescents in this study aged 12–18 years (*M* = 15.39, SD = 1.81), and 114 were female (72.6%). The majority was ethnic Han (96.8%). Among them, 134 patients came from urban area (85.4%), 30 patients were troubled by romantic relationship (19.1%), 32 patients' parents were divorced (20.4%). There were no differences between the two groups in age, gender, residence, nationality, parent's marriage, educated years. However, there was significant difference in troubled by romantic relationship between NSSI group and non-NSSI group (χ^2^ = 5.38, *P* = 0.02).

Among them, 103 (65.6%) patients were from outpatient department, 62 (39.5%) patients were not first-episode. There were significant differences in first-episode (χ^2^ = 4.37, *P* = 0.03), hospitalization times (*t* = 3.60, *P* < 0.001), on mood-stabilizer treatment (χ^2^ = 8.53, *P* < 0.001), SAG PDD/DDD value (*t* = 3.64, *P* < 0.001) and benzodiazepine PDD/DDD value (*t* = 3.46, *P* < 0.001) between NSSI group and non-NSSI group. There was no significant difference in antidepressant PDD/DDD between two groups.

In terms of adverse life events, Table 1 shows mean score of CTQ-SF in the NSSI group was 52 (SD = 12.72), while adolescents without NSSI was 44.26 (SD = 11.47). The NSSI group experienced a greater incidence of childhood traumatic events than the other (*t* = 3.99, *P* < 0.001). As far as subscales were concerned, we observed significant differences in all except the subscale SA. The mean score of OBVQ-R in NSSI was 6.94 (SD = 6.79), while in the non-NSSI group was 3.32 (SD = 4.00). The results demonstrated greater suffering in patients composing the NSSI group in peer bullying than patients outside of it (*t* = 4.07, *P* < 0.001).

In terms of clinical symptoms, Table 1 shows NSSI group having higher scores on MADRS (mean = 52.00, SD = 12.72) and BPRS (mean = 29.34, SD = 7.76) with significant difference. There was no significant difference in YMRS total score between two groups.

Table 2 shows M.I.N.I. diagnosis of this sample. Suicidality (current) (60.5%), major depressive disorder (current) (59.2%), major depressive disorder (recurrent) (28.0%), manic episode (recurrent) (27.4%) and social phobia (current) (16.6%) were most common. There were significant differences in suicidality (current) (χ^2^ = 14.33, *P* < 0.001) and major depressive disorder (current) (χ^2^ = 9.75, *P* = 0.002) between NSSI group and non-NSSI group.

**Table 2:** M.I.N.I. diagnosis of NSSI group and Non-NSSI group.

	**Overall (*****n*** **=** **157)**		**NSSI (*****n*** **=** **80)**		**Non-NSSI (*****n*** **=** **77)**		**Statistics**		
	***N***	**%**	***N***	**%**	***N***	**%**	***X* ^2^**	**df**	***P***
Major depressive disorder (Current)	93	59.2	57	71.3	36	46.8	9.75	1	**0.002**
Major depressive disorder (Recurrent)	44	28.0	22	27.5	22	28.6	0.22	1	0.88
Dysthymia (Current)	10	6.4	4	5.0	6	7.8	0.51	1	0.47
Suicidality (Current)	95	60.5	60	75.0	36	45.5	14.33	1	**<0.001**
Manic episode (Current)	9	5.7	6	7.5	3	3.9	0.94	1	0.33
Manic episode (Recurrent)	43	27.4	23	28.8	20	26.0	0.15	1	0.69
Panic disorder (Current)	18	11.5	9	11.3	9	11.7	0.007	1	0.93
Panic disorder (Lifetime)	7	4.5	4	5.0	3	3.9	0.11	1	0.73
Agoraphobia (Current)	5	3.2	2	2.5	3	3.9	0.24	1	0.61
Social phobia (Current)	26	16.6	15	18.8	11	14.3	0.56	1	0.45
Obsessive-compulsive disorder (Current)	16	10.2	10	12.5	6	7.8	0.95	1	0.33
Posttraumatic stress disorder (Current)	11	7.0	8	10.0	3	3.9	2.24	1	0.13
Alcohol dependence (Current)	0	0	–	–	–	–	–	–	–
Substance dependence (Current)	0	0	–	–	–	–	–	–	–
Psychotic disorder (Lifetime)	11	7.0	7	8.8	4	5.2	0.76	1	0.38
Psychotic disorder (Current)	4	2.5	4	5.0	0	0	2.19	1	0.13
Anorexia nervosa (Current)	1	0.6	0	0	1	1.3	0	1	0.98
Bulimia nervosa (Current)	3	1.9	2	2.5	1	1.3	0	1	1
Generalized anxiety disorder (Current)	39	24.8	20	25.0	19	24.7	0.002	1	0.96
Antisocial personality disorder (Lifetime)	4	2.5	3	3.8	1	1.3	0.95	1	0.33

*Bold values: P < 0.05; M.I.N.I., The Mini-International Neuropsychiatric Interview*.

Table 3 illustrates the factors in socio-demographic, clinical and psychological leading to NSSI. Stepwise logistic regression analysis for the prediction of NSSI in the last year included the following indicators: age, gender, troubled by romantic relationship, MADRS total score, OBVQ-R total score, and CTQ-SF dimensions. Finally, the significant factors included troubled by romantic relationship [Odds ratio (OR) = 4.28, 95% confidence interval (CI) = 1.36–13.44], OBVQ-R total (OR = 1.11, 95% CI = 1.04–1.19), MADRS total (OR = 1.05, 95% CI = 1.01–1.09). The regression model explained 24.3% of the registered variance.

**Table 3:** Socio-demographic, clinical and psychological correlates of NSSI (two binary logistic regression analysis).

	**OR**	**95% C.I**.	***P***
Trouble in romantic relationship	4.28	1.36–13.44	**0.013**
OBVQ-R total	1.11	1.04–1.19	**0.002**
MADRS total	1.05	1.01–1.09	**0.003**

*Bold values: P < 0.05; OBVQ-R: the revised Olweus bully/victim questionnaire; 95% CI,: 95% confidence interval; MADRS, montgomery-asberg depression rating scale; OR, odds ratio; Likelihood ratio: 185.97, R^2^ = 0.243*.

Table 4 shows the Pearson's correlations between NSSI overall severity with CTQ-SF total score, all of the CTQ-SF subscales' score, OBVQ-R total score and MADRS total score among NSSI group.

**Table 4:** Pearson's correlations between NSSI overall severity and psychological traits, depression among NSSI group (*n* = 80).

**Factors**	**NSSI overall severity**
CTQ-SF total	0.455^**^
Emotional abuse	0.448^**^
Physical abuse	0.331^**^
Sexual abuse	0.244^**^
Emotional neglect	0.259^**^
Physical neglect	0.231^*^
OBVQ-R total	0.492^**^
MADRS total	0.243^**^

*OBVQ-R, the revised olweus bully/victim questionnaire; CTQ-SF, childhood trauma questionnaire-short form; MADRS, montgomery–asberg depression rating scale*.

* *p < 0.05;*

** *p < 0.01*.

## Discussion

The aim of this study was to explore influencing factors and clinical characteristics of NSSI in adolescent with mental distress. There were several main findings in this study. Adolescent patients with NSSI experienced more adverse life events including peer bullying, childhood trauma and romantic relationship trouble. They had more times of hospitalization, higher dosage of psychotic medication, and more severe depressive symptoms.

There were a lot of studies exploring the complexity and diversity of risk factors on NSSI (35). Our finding was consistent with a previous study about an integrated theoretical model of NSSI (36), showing that the risk of NSSI increased with the presence of distal factors (e.g., childhood maltreatment) and the current stressful life events (e.g., peer bullying, family relationships, romantic relationships).

Previous studies have also shown that overall childhood maltreatment (CT) was associated with NSSI (37, 38). Nock (36) reported that CT led to intrapersonal vulnerability factors (e.g., high aversive emotions and cognitions, poor distress tolerance) and interpersonal vulnerability factors (like poor communication skills and meager social problem-solving). However, there were inconsistent findings concerning the association between childhood trauma domains and NSSI. For example, CT and its subtypes were associated with NSSI, excepting childhood emotional neglect in one study (38). Interestingly, Serafini et al. (39) reported a direct link of CT, especially childhood sexual abuse, with NSSI. However, we found that adolescents with NSSI encountered more frequently traumatic experiences except for SA in this study. Different sample sizes and control of confounding factors may account for the inconsistent results. In addition, teenagers are perhaps reluctant to talk about traumatic experience, especially sexual abuse, which led the difficulty in the psychological intervention.

In this study, peer bullying was a significant factor of NSSI in adolescent psychiatric patients and was consistent with many previous studies. Van Geel et al.'s meta-analytic study (40) found that peer victimization was one of the major correlations to NSSI during adolescence, showing that the odds of NSSI were higher by 2.1 times in victims compared to children who did not face bullying in school. Previous study suggested NSSI as a maladaptive coping mechanism to deal with negative emotions after a bullying incident (41, 42). Moreover, it has been proposed that intrapersonal factors, such as self-compassion (43) and depression (44) moderate the relationship between the incidence of being bullied and NSSI. It is noteworthy to mention a prior study (45), which indicated that psychological mechanisms such as impulsivity, reduced self-regulating abilities were the underlying association between being bullied and NSSI.

Trouble in romantic relationship was also a significant risk factor for NSSI in this study. In another study, they also found the similar result with us that only negative romantic relationship events were significantly associated with the risk of current NSSI (46).

Previous study showed several interventions appear to hold promise for reducing NSSI, including psychological therapy (e.g., dialectical behavior therapy, emotion regulation group therapy etc.), atypical antipsychotics (e.g., aripiprazole), and selective serotonin reuptake inhibitors. Nevertheless, there remains a paucity of well-controlled studies investigating treatment efficacy for NSSI (47).

In this study, adolescent psychiatric patient with NSSI was admitted to the hospital more repetitively, which indicated the difficulty of treatment and management. Furthermore, they were on more dosage of benzodiazepine, second generation antipsychotics and mood-stabilizer drugs. A study indicated individual with NSSI having severe impulsivity (48), which may explain the higher use of dosage of psychotic drugs, especially the use of mood stabilizer, benzodiazepine, and SGAs in treating patients with NSSI. Thus, it still needs further exploration about the balance of benefit from the use of psychotic medication and the adverse side effects on adolescent psychiatric patients with NSSI.

Severe depressive symptoms was a significant factor related to NSSI in this study, which was consistent with one previous study (49). Among patients with depression, individuals experience more negative emotions (50, 51) which increased the risk of NSSI due to deficit of emotion regulation (52). Furthermore, a study conducted during COVID-19 pandemic suggest that psychiatric patients were at higher levels of symptoms of depression (53).

From the perspective of clinical implications based on the main findings in this study, the in-depth evaluation of trauma experience and adverse life events such as peer bullying and romantic relationship trouble is of importance for timely detection and targeted treatment of NSSI. The intervention of those adverse life events and trauma experience could perhaps be a breakthrough of psychological therapy in these adolescent patients with NSSI. Meanwhile, the effective treatment of depressive symptoms will also contribute to the improvement of NSSI.

### Strengths and Limitations

This study has the following advantages. Firstly, all participants filled in the case report form and received face-to-face interviews, which greatly improved the reliability of the data source. Secondly, our samples came from the clinical population. This would help to have a deeper understanding of NSSI, the factors related to NSSI, and provided the worthy treatment strategies in the clinical practice concerning the patients with NSSI.

This study also faces some limitations. Because of the cross-sectional design of this study, the results reflect the correlation of risk factors rather than causality. All participants were from a mental health institution in China and the results of this study was perhaps not applicable to other mental health institutions.

## Conclusions

It is very necessary to evaluate negative life events, including childhood trauma, peer bullying etc. in adolescent patients with NSSI. It has important clinical implications for evaluating the risk factors of NSSI and giving effective intervention treatment. Timely and effective treatment of these patients' depressive symptoms will also contribute to the relief of NSSI.

## Data Availability Statement

The original contributions presented in the study are included in the article/supplementary files, further inquiries can be directed to the corresponding author/s.

## Ethics Statement

The studies involving human participants were reviewed and approved by the Ethics Committees of Guangdong Provincial People's Hospital. Written informed consent to participate in this study was provided by the participants' legal guardian/next of kin.

## Author Contributions

C-LH and J-JZ designed the study and analyzed and interpreted the patient data. J-JZ was a major contributor in writing the manuscript. J-JZ, Y-DL, Z-HH, and HZ were responsible for sample collection. FW and J-JY made the psychological evaluation. S-BW and FW participated in statistical analysis of data and prepared the tables. All authors read and approved the final manuscript.

## Funding

This work was supported by Guangdong Provincial Foundation for Basic and Applied Basic Research Natural Science Foundation (grant number: 2022A1515010619).

## Conflict of Interest

The authors declare that the research was conducted in the absence of any commercial or financial relationships that could be construed as a potential conflict of interest.

## Publisher's Note

All claims expressed in this article are solely those of the authors and do not necessarily represent those of their affiliated organizations, or those of the publisher, the editors and the reviewers. Any product that may be evaluated in this article, or claim that may be made by its manufacturer, is not guaranteed or endorsed by the publisher.
